# Society Meetings

**Published:** 1890-06

**Authors:** 


### Editorial.

### SOCIETY MEETINGS.

The Illinois State Dental Society held its twenty-sixth annual session at Springfield, May 13 to 16, inclusive. This society is one of the oldest in the United States, being, however, two years junior to the Iowa State Society, which held its regular meeting one week previous. The Illinois State Society has been considered one of the best working societies in the country, and this session was no exception to the rule. It was six years since it had been our pleasure to meet with this body, and we were impressed with certain changes that had come over it. The St. Louis delegation used to be out in full force. It was, however, noticeable this year for its absence. The Ohio delegation, led by Drs. Taft and Smith, also failed to put in an appearance. The meeting was one composed largely of home talent, and not all of that was out, Drs. Allport, Marshall, and Talbott being conspicuous by their absence. Nevertheless, the meeting was a good one and fully in keeping with former years. The papers were good, but, with one exception, there was a dearth of interest in their discussion. The clinics were numerous and up to the usual in interest. It was a scientific meeting, and many remarked the lack of interest in the display of dental goods. The S. S. White Dental Manufacturing Company had no stock on exhibit. The remark was common that the dealers did not sell enough to pay their expenses.

The Society numbers one hundred and forty-three active members, with only one death during the past year, that of Dr. S. M. Sturgis, of Quincy.

The forenoon was taken up by the discussion of the proposed change in the constitution and by-laws, which contemplated vesting in an executive council, composed of nine members and the president, secretary, and treasurer, the entire business of the society, thus leaving the time of the main body free for the consideration of scientific questions. This move was strenuously opposed by Drs. Stevens, C. Stoddard Smith, and A. E. Mattison as undemocratic and tending to centre too much power in the hands of a few individuals. The measure was finally adopted as proposed, and hereafter all business, including the nomination of the officers and the choosing of the place of meeting for the following year, will be left to the council, which is to be geographically distributed over the State.

The members of the council are C. P. Pruyn and George H. Cushing, Chicago; J. W. Cormany, Mt. Carroll; K. B. Davis, Springfield; Charles Henry, Jacksonville; J. J. Jennelle, Cairo; W. T. Magill, Rock Island; J. D. Moody, Mendota; W. H. Taggart, Freeport. This is a move in the right direction if the council can be kept free from political rings. The time of many societies is often taken up by business to such an extent that little is left for the consideration of scientific subjects.

The president, Dr. Thomas W. Pritchett, of Whitehall, spoke in fitting terms in his address of the individual work of the members of the society since its organization in the direction of higher education, which he said was fast becoming an accomplished fact.

Dr. Thomas L. Gilmer, Chicago, gave a clear and logical review of the past year's work in dental science and literature. In discussing this paper, Dr. J. J. R. Patrick, Belleville, made a characteristic speech, claiming that all dental societies should withdraw the publication of their proceedings from journals published by manufacturers of dental goods.

Dr. G. V. Black spoke interestingly on the crania of the mound-builders and early Indians. Dr. Patrick objected to the distinction made by Dr. Black, holding that they were one and the same race and lived at the same time.

Dr. S. Finley Duncan read an interesting paper relating to higher education, deprecating the abuse of colleges advertising in an unprofessional manner for patients and students, claiming that it had a bad effect upon the latter.

Dr. Sudduth occupied one evening session of the society with an illustrated lecture on oral pathology, confining his attention to the micro-organisms found in the oral cavity.

Dr. Black, in opening the discussion, spoke appreciatively of the character of the work presented, and approved of the special manner of illustrating a most difficult subject to large classes.

Dr. J. J. R. Patrick read a scholarly paper on "The Second Period of Dentistry," which was listened to with the closest attention. Dr. Patrick is a close student, and his writings are generally of a character that will live. If more men in the profession wrote from the same stand-point, dental literature would have a less evanescent existence.

The society elected Dr. Brophy as president, and adjourned to meet in Bloomington, Ill., the following year.

